# How to balance valuable innovation with affordable access to medicines in Belgium?

**DOI:** 10.3389/fphar.2022.960701

**Published:** 2022-09-16

**Authors:** Steven Simoens, Khadidja Abdallah, Liese Barbier, Teresa Barcina Lacosta, Alessandra Blonda, Elif Car, Zilke Claessens, Thomas Desmet, Evelien De Sutter, Laurenz Govaerts, Rosanne Janssens, Teodora Lalova, Evelien Moorkens, Robbe Saesen, Elise Schoefs, Yannick Vandenplas, Eline Van Overbeeke, Ciska Verbaanderd, Isabelle Huys

**Affiliations:** ^1^ KU Leuven Department of Pharmaceutical and Pharmacological Sciences, Leuven, Belgium; ^2^ KU Leuven Centre for IT & IP Law (CiTiP), Leuven, Belgium; ^3^ European Organisation for Research and Treatment of Cancer, Brussels, Belgium; ^4^ Anticancer Fund, Strombeek-Bever, Brussels, Belgium

**Keywords:** innovation, access, affordability, medicines, Belgium

## Abstract

**Background:** Countries are struggling to provide affordable access to medicines while supporting the market entry of innovative, expensive products. This *Perspective* aims to discuss challenges and avenues for balancing health care system objectives of access, affordability and innovation related to medicines in Belgium (and in other countries).

**Methods:** This *Perspective* focuses on the R&D, regulatory approval and market access phases, with particular attention to oncology medicines, precision medicines, orphan medicines, advanced therapies, repurposed medicines, generics and biosimilars. The authors conducted a narrative review of the peer-reviewed literature, of the grey literature (such as policy documents and reports of consultancy agencies), and of their own research.

**Results:** Health care stakeholders need to consider various initiatives for balancing innovation with access to medicines, which relate to clinical and non-clinical outcomes (e.g. supporting the conduct of pragmatic clinical trials, treatment optimisation and patient preference studies, optimising the use of real-world evidence in market access decision making), value assessment (e.g. increasing the transparency of the reimbursement system and criteria, tailoring the design of managed entry agreements to specific types of uncertainty), affordability (e.g. harnessing the role of generics and biosimilars in encouraging price competition, maximising opportunities for personalising and repurposing medicines) and access mechanisms (e.g. promoting collaboration and early dialogue between stakeholders including patients).

**Conclusion**: Although there is no silver bullet that can balance valuable innovation with affordable access to medicines, (Belgian) policy and decision makers should continue to explore initiatives that exploit the potential of both the on-patent and off-patent pharmaceutical markets.

## Introduction

Health care stakeholders in Belgium and across the globe are challenged to balance access to innovative medicines against issues of financial sustainability and equity (European Medicines Agency, 2018; Oortwijn et al., 2018; Maes et al., 2019; Facey et al., 2020). The emergence of highly innovative yet expensive medicines puts strain on pharmaceutical budgets. For instance, public pharmaceutical expenditure in Belgium grew annually by around 6% from 2016 onwards, and amounted to € 5.2 billion in 2019 (National Institute for Health and Disability Insurance, 2020b). Medicine classes accounting for a large proportion of public pharmaceutical expenditure in 2019 included other antineoplastic agents (19.6%) and immunosuppressants (14.8%).

Market access of medicines is a crucial factor in improving population life expectancy and quality of life. For instance, an analysis of the health effect of pharmaceutical innovation calculated that medicines which received marketing authorisation between 1987 and 1995 decreased the premature cancer mortality rate by 20% and added 1.52 years to the mean age at death from cancer in Belgium in 2012 (Lichtenberg, 2016). Improved life expectancy and quality of life, in turn, increase labour productivity. A recent study for example showed that although the market access of innovative curative medicines for hepatitis C significantly increased pharmaceutical expenditure, this increase was more than offset by savings arising from less use of other medicines, avoidance of cirrhosis and further contamination, and increased productivity in Belgium (SEBOIO, 2020).

The challenge of guaranteeing affordable access to medicines is also highlighted in the 2020 policy plan of the Belgian Minister of Health (Vandenbroucke, 2020). It is the intention of the Minister, amongst other things, to agree a new pact with pharmaceutical industry, which not only aims to sustain innovation and access to medicines, but will also include measures to control pharmaceutical expenditure and to address the budgetary responsibility of the industry.

Balancing valuable innovation with affordable access to medicines in Belgium has been rendered more difficult by the COVID-19 pandemic. In response to this crisis, the Belgian government has made available €2 billion to guarantee continuity of care in hospitals and to compensate them for the loss of income due to the postponement of care. According to data pertaining to the first semester of 2020, expenditure on physician consultations exhibited a sizeable decrease, but pharmaceutical expenditure continued to grow in Belgium (National Institute for Health and Disability Insurance, 2020c). However, the COVID-19 pandemic also taught us that market access of innovative technologies (like the new mRNA vaccines) highly depends on citizens’ and patients’ willingness to accept the technology (Coustasse et al., 2021). Patient involvement in market access of medicines is therefore crucial.

**FIGURE 1:**
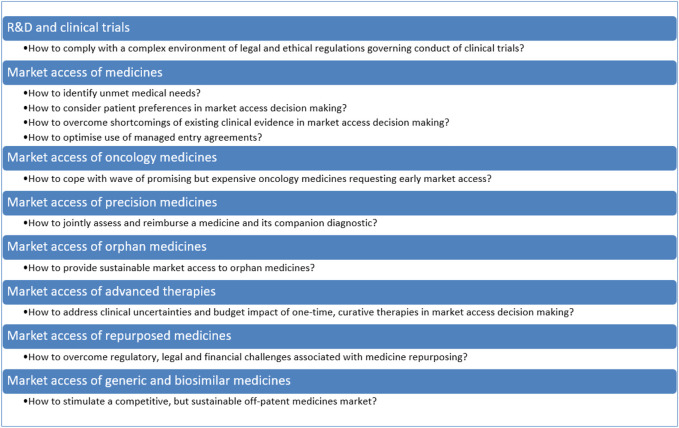
Challenges in balancing valuable innovation with affordable access to medicines.

**FIGURE 2:**
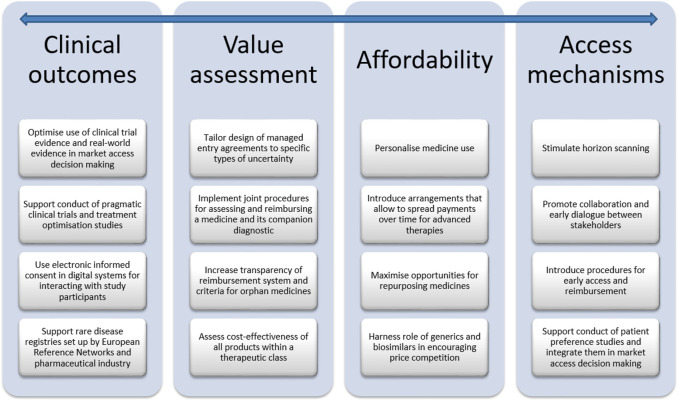
Avenues for balancing valuable innovation with affordable access to medicines.

The aim of this *Perspective* is to explore challenges and avenues related to clinical outcomes, value assessment, affordability and access mechanisms for balancing valuable innovation with affordable access to medicines. To this effect, a narrative review was undertaken of the peer-reviewed literature and of the grey literature, including policy documents, legal texts, reports of consultancy agencies and position statements. This *Perspective* also drew on the 15 years of experience that the research team has in investigating regulatory aspects of market access of medicines in Belgium. Although this manuscript pertains specifically to Belgium, many of the avenues discussed are also being investigated in other countries and are relevant to an international audience. The manuscript also refers to international initiatives related to market access of medicines when they are relevant to Belgium. The manuscript is structured according to the different phases of the life cycle of medicines, from R&D, market access of innovative medicines in general and of specific classes (i.e., oncology medicines, personalized medicines, orphan medicines, advanced therapies), to repurposed medicines, generic and biosimilar medicines. The main challenges and avenues for balancing valuable innovation with affordable access to medicines are summarised in Figure 1 and Figure 2, respectively, and are discussed in more detail in the following sections.

## R&D of medicines: Focus on clinical trials

Innovation is driven by R&D and biopharmaceutical companies invested €4.96 billion in R&D in Belgium in 2020 (Pharma be, 2021a). Given that Belgium plays a major role in hosting clinical trials of medicines (Flanders Investment & Trade, 2021) - for instance, 503 clinical trial applications were approved in 2020 (Pharma be, 2021b) - this section focuses on recent developments in the clinical trial landscape, namely the (new) legal landscape governing clinical trials, the role of pragmatic trials, and the (upcoming) implementation of electronic informed consent.

### A new complex legal landscape on clinical trials

In Belgium, the conduct of clinical trials is governed by the Law of 7 May 2004 concerning experiments on the human person, which was put in place to implement the EU Clinical Trials Directive (EC) 2001/20/EC. However, the Clinical Trials Directive - criticised for deterring research (Rice, 2004) - has been replaced by the Clinical Trials Regulation (EU) 536/2014 on 31 January 2022 (European Medicines Agency, 2014a). The Clinical Trials Regulation aims to strengthen harmonisation in the regulation of interventional clinical trials, and in particular to stimulate the conduct of pan-European trials (European Medicines Agency, 2014a). At the same time as the Clinical Trials Regulation, the new Belgian Law of 7 May 2017 concerning experiments with investigational medicinal products in humans has also entered into force. Belgium has been actively preparing for the new rules, as evidenced by the 2020 policy document of the Minister of Health (Vandenbroucke, 2020).

The Clinical Trials Regulation introduces a number of novel provisions. It is meaningful to highlight one example, namely the definition of “low-intervention trials”. In particular, a low-intervention trial means a trial that studies an authorised investigational medicinal product, and in which the product is used in accordance with the terms of the marketing authorisation or its use is evidence-based and supported by published scientific evidence on safety and efficacy. The additional diagnostic or monitoring procedure should not pose more than minimal additional risk or burden to the safety of the study subjects, compared to normal clinical practice (European Medicines Agency, 2014a). Low-intervention clinical trials benefit from a more lenient regime of obligations, compared to interventional clinical trials, e.g. as regards submission of application, obtaining informed consent, and monitoring. The establishment of the definition of low-intervention trials is of particular interest, as it recognises the importance of pragmatic trials for clinical research (see “2.2 Opportunity for pragmatic clinical trials in Belgium?“) (Musch, 2017).

In addition to the Clinical Trials Directive / Clinical Trials Regulation and the implementing Belgian laws, a large number of other legal and ethical instruments have to be complied with (such as rules governing biobanking, medical devices and *in vitro* medical devices), and not all of them were designed with the goal to be applied simultaneously, as it may be necessitated by the demands of for example precision medicine (Negrouk et al., 2018). Moreover, the principles of the EU General Data Protection Regulation 2016/679 have to be complied with in all cases when personal data is processed for research. The General Data Protection Regulation aims to protect individuals with regard to the processing of personal data, and to facilitate the free movement of such data. However, the General Data Protection Regulation also introduces challenges for the conduct of health research and clinical trials in particular (Negrouk and Lacombe, 2018; van Veen, 2018; Lalova et al., 2020). One challenge relates to the secondary use of personal data for health research, especially when it comes to the respect of the transparency obligations towards study participants. For this, digital systems for interacting with participants including electronic informed consent (see “2.3 Electronic informed consent”) may offer value.

### Opportunity for pragmatic clinical trials in Belgium?

The Clinical Trials Regulation’s introduction of the concept of low-intervention trials could have major implications for the conduct of pragmatic clinical trials, which aim to evaluate how well a particular health technology works under real-life circumstances (Ford and Norrie, 2016). In many respects, pragmatic trials can be considered low-interventional in nature (Dal-Re et al., 2017; Dal-Re et al., 2019), since they are typically designed to measure and compare the effectiveness of already approved products that are administered within the scope of their marketing authorisations under conditions that reflect real-world clinical practice. Although patients may benefit from the outcomes of pragmatic trials, from a commercial point of view, no financial gains are to be expected as the products used in the trials are already on the market. The more lenient obligations imposed by the Clinical Trials Regulation for low-intervention trials (European Medicines Agency, 2014a) could though facilitate and stimulate the set-up of pragmatic clinical trials in Belgium, which until now have had to abide by the same set of stringent regulatory requirements applicable to any other clinical trial. Due to the limited interest of the commercial sector in such research, pragmatic clinical trials have mainly been undertaken independently by academic stakeholders, who have faced difficulties in securing the resources necessary to perform legally compliant clinical studies of a sufficient size to produce meaningful results (Neyt et al., 2016; Nevens et al., 2019). In an effort to support academia-affiliated researchers in conducting pragmatic trials with a high potential of generating cost savings for the health care system, the Belgian government has since 2016 been funding non-commercial and practice-oriented clinical research projects through calls launched by the Belgian Health Care Knowledge Centre (Belgian Health Care Knowledge Centre, 2021). So far, 42 studies have been initiated under its Trials programme, recruiting more than 21,000 adult and pediatric patients across a multitude of different disease areas, including COVID-19. Besides the limited number of structural funding mechanisms available for pragmatic trials, one of the other challenges in running such studies lies in the participants’ ability to provide their informed consent (Kalkman et al., 2017). Because they are situated at the interface between clinical research and routine care, pragmatic trials require alternative informed consent procedures that are compatible with their objectives and methodology (e.g. to accommodate the use of cluster randomisation), such as electronic informed consent (McKinney et al., 2015).

### Electronic informed consent

The 2020 policy document of the Belgian Minister of Health (Vandenbroucke, 2020) highlights the importance of investigating digital technologies, such as electronic informed consent, to support compliance with the Good Clinical Practice standard. According to this standard, study participants need to receive oral and written information about all pertinent aspects of a study during the informed consent process to enable an informed decision on study participation (European Medicines Agency, 2016). Digitalizing informed consent provides numerous advantages (De Sutter et al., 2020). With electronic informed consent, participants can indicate their preferences to engage in clinical research over time. In addition, electronic informed consent enables the research team to communicate more effectively with participants during and after a study. Moreover, participants can choose to receive research outcomes, which may foster transparency in clinical research. An interactive electronic informed consent system may further improve transparency by offering an overview of the use of participants’ data across research studies, which could be a potential solution for challenges posed by the General Data Protection Regulation (Kaye et al., 2015; Budin-Ljosne et al., 2017). At a Belgian level, empirical literature regarding electronic informed consent is scarce (De Sutter et al., 2021). Also, the Clinical Trial College, a Belgian governmental body, coordinated the development of guidance related to the use of electronic informed consent in interventional clinical trials (Clinical Trial College, 2021; Federal Public Service Health Food Chain Safety and Environment, 2021). At European level, the European Medicines Agency drafted a new guideline, aiming to support stakeholders to comply with the current legal landscape when using computerized systems, including electronic informed consent, in clinical trials (European Medicines Agency, 2021b).

## Market access of medicines

### Context and challenges

Belgian (and other European) health care authorities and policy makers face numerous challenges related to the market access of medicines in general. For instance, there is a lack of harmonised, international approaches for systematically identifying unmet medical needs across therapeutic areas (European Commission, 2020). In the context of a demand-driven system of medicine development, such approaches are important to mitigate the risk of granting access to medicines that are not addressing unmet medical needs, and hence, have little impact in clinical practice (Cutler et al., 2018). Belgium has in place a procedure for granting early treatment access and reimbursement to innovative medicines for unmet medical needs, but this procedure is currently under revision (Vandenbroucke, 2020; National Institute for Health and Disability Insurance, 2021b). One key issue is the lack of a clear definition of unmet medical needs, as this term is used in different ways in several regulatory and legal instruments at the regulatory approval stage (e.g., orphan drug regulation, conditional market authorisation regulation) as well as market access stage (e.g., health technology assessment and reimbursement criterion in different European countries). For instance, the Belgian Health Care Knowledge Centre performed a pilot study on the systematic identification of unmet medical needs in the form of a multi-criteria decision tool (Cleemput et al., 2016). There is also a lack of clear procedures to anticipate the market entry of new medicines with a view to assessing their impact on the sustainability of expenditure (Oortwijn et al., 2018).

Further, integrating societal or patient perspectives in market access has been considered important, yet not implemented. One interesting type of patient perspectives are patient preferences, relating to choices of patients towards which treatment characteristics matter to them, why, to what extent and which trade-offs play. Pharmaceutical industry and health care decision makers have called for methodologies that enable them to measure and integrate patient preferences in medicine research and decision making (Medical Device Innovation Consortium, 2015; Johnson and Zhou, 2016; Vandenbroucke, 2020). Patient preferences can be used to inform the selection and assessment of unmet treatment needs, the treatment outcomes (benefits, risks) and uncertainties related to these outcomes (e.g., regarding their long-term duration and severity). However, approaches and the impact of incorporating patient preferences during medicines development and assessment is presently unsystematic, low and scattered across different phases of the lifecycle of medicines (Janssens et al., 2018; Hansen et al., 2019). Moreover, an array of challenges related to patient preference studies need to be further investigated, including: 1) the need for a systematic and robust preference study methodology; 2) the need for unbiased patient preference studies; 3) insights into how to deal with preference heterogeneity in studies; and 4) insights about whether preference studies need to be designed towards a single medicine or need to be product-“agnostic” (Medical Device Innovation Consortium, 2015; Utens C. et al., 2015; Ho et al., 2016).

Market access is also challenged by limitations of available clinical evidence (e.g., about the durability of clinical effects) that informs marketing authorisation and reimbursement of innovative medicines (Oortwijn et al., 2018; Facey et al., 2020; Eichler et al., 2021). Such evidence gaps translate into uncertainty concerning the (long-term) efficacy, safety and cost-effectiveness of the new medicine. Scenarios of increased uncertainty are especially present when medicines need to be developed, authorised and reimbursed in a limited time frame; when there are high unmet needs and early access is desired, such as during the COVID-19 pandemic. Clinical evidence generation has additionally been criticised for not systematically including patient-relevant outcomes (van Overbeeke et al., 2019a; van Overbeeke et al., 2019b).

Innovative medicines characterised by clinical uncertainties and a high budgetary impact are increasingly subjected to managed entry agreements (Zampirolli Dias et al., 2020). Their implementation in Belgium has generated substantial savings (i.e., compensation of 38.5%, amounting to € 1.6 billion gross turnover in 2019) (National Institute for Health and Disability Insurance, 2020b)*.* However, a recent review of Belgian managed entry agreements criticised the lack of a clear link between identified uncertainties and the type and content of the agreement, and the limited transparency of the pricing and reimbursement system (Neyt et al., 2020). With respect to the latter, a coalition of three not-for-profit or non-governmental organisations has asked for an independent public authority to be granted access to and evaluate managed entry agreements for medicines in Belgium (Dokters van de Wereld, 2018).

### Sustaining innovation and access to medicines

In the context of identifying unmet medical needs and innovative health technologies, Belgium participates in the International Horizon Scanning Initiative (Lepage-Nefkens et al., 2017; National Institute for Health and Disability Insurance, 2021a). This initiative focuses on medicines putting significant pressure on pharmaceutical expenditure and/or with a highly innovative character. The first important output (expected in 2025) will be a database of all publicly available information about medicines in the pipeline. This database aims to enable governments to better anticipate innovative treatments, prioritise pharmaceutical expenditure and identify areas for which insufficient treatments are available (and, hence, unmet medical needs exist).

To anticipate market entry of innovative medicines, the Belgian government has also stipulated to strengthen its voluntary collaboration with the Netherlands, Luxembourg, Austria and Ireland in the BeNeLuxA Initiative on Pharmaceutical Policy (Vandenbroucke, 2020). In this initiative, reimbursement authorities of these countries work together to support sustainable access to innovative medicines by means of horizon scanning, health technology assessment, information sharing and joint price negotiations (BeNeLuxA Initiative on Pharmaceutical Policy, 2021). Although BeNeLuxA initially focused on orphan medicines (e.g. for cystic fibrosis and spinal muscular atrophy), its scope has been extended to medicines generating a high budget impact (e.g. advanced therapies). To date, the success of BeNeLuxA has been hampered by the need to invest resources in setting up an operational structure and supporting legal framework, the need to align pricing and reimbursement procedures, a lack of transparency and clarity on how the BeNeLuxA procedures work, and the limited willingness of pharmaceutical companies to submit applications (Vogler et al., 2021), since advantages of market access via BeNeLuxA remain unclear for companies.

Also, in the domain of inclusion of patient perspectives in market access, limited but important steps forward in Belgium have been taken by the participation of a patient in the commission tasked with evaluating clinical trials and advising the Minister on the national marketing authorisation; the presence of patient representatives during reimbursement discussions; and the organisation of citizen meetings to gather their views on the relevance of reimbursement decision criteria to rank unmet medical needs. In view of a more structured approach, it is also worth mentioning the European Innovative Medicines Initiative PREFER. This ongoing initiative (end in 2022) involves pharmaceutical industry, regulators and health technology assessment agencies (including the Belgian Health Care Knowledge Centre) and works towards best practices for patient preference studies and guidelines on how to design, conduct, analyse and use such studies (Innovative Medicines Initiative, 2021). Based on the insights of the PREFER project, the European Medicines Agency (along EUnetHTA) provided a positive qualification opinion on a framework for measuring and using patient preferences as one type of patient input into decision-making (European Medicines Agency, 2021a).

The European Medicines Agency and EUnetHTA are also collaborating on joint requirements to enable better clinical evidence generation (European Medicines Agency, 2018; European Medicines Agency, 2020). Specifically, these organisations strive to develop a single evidence-generation plan and to perform joint parallel consultations with a view to help generate clinical evidence that satisfies the needs of both marketing authorisation and reimbursement decision making (Hines et al., 2020a; Hines et al., 2020b; European Medicines Agency, 2020). Areas of collaboration include, for example, sharing methodological approaches for the design, analysis and interpretation of clinical trials and observational studies (European Medicines Agency and EUnetHTA, 2021).

In order to manage uncertainties due to gaps in clinical trial evidence, there is increased consensus regarding the need to use clinical trial evidence *and* real-world evidence for the assessment of safety and (cost-)effectiveness of medicines (Facey et al., 2020; Eichler et al., 2021). Therefore, the Belgian National Institute for Health and Disability Insurance convened a series of multi-stakeholder roundtables, which resulted in the proposal of guiding principles for the use of real-world evidence in market access (Annemans, 2017). Furthermore, the RWE4Decisions initiative (also supported by the National Institute for Health and Disability Insurance) has called for international co-operation to decide on real-world evidence requirements throughout a medicine’s lifecycle, transparency in the generation of real-world evidence, and the development of analytical methods to use real-world evidence in health technology assessment (Facey et al., 2020).

Additionally, the existence of clinical uncertainties has led to the implementation of new pricing and reimbursement approaches such as outcome-based managed entry agreements (Maes et al., 2019; Neyt et al., 2020). The importance of outcome-based assessment was the topic of discussion in a series of roundtable discussions set up by the National Institute for Health and Disability Insurance in 2016, resulting in recommendations to advance outcome-based pricing and reimbursement of innovative medicines (Annemans and Pani, 2016). Outcome-based reimbursement seems to be particularly relevant to support market access of advanced therapies (see “7. Advanced therapies”).

## Market access of oncology medicines

### Context and challenges

Challenges related to oncology medicines have been amply discussed in the literature, but include an extensive pipeline of oncology medicines in development, expedited marketing authorisation based on immature clinical evidence, the market entry of expensive immunotherapies for a multitude of indications, uncertainty about the size of health gain generated by innovative oncology medicines, increasing oncology medicine prices over time, concerns about the cost-effectiveness of these products, and questions surrounding their real-life use (e.g. how to combine and sequence new oncology medicines with existing therapies) (Van Dyck et al., 2016; Wilking et al., 2017; Saesen et al., 2020; IQVIA Institute for Human Data Science, 2021; Neyt et al., 2021). Oncology medicines also encompass a plethora of different product types, including precision medicines, orphan medicines and advanced therapies, with each type exhibiting specific market access challenges which are discussed in separate sections in this manuscript.

To illustrate the challenging context surrounding market access of oncology medicines in Belgium, the National Institute for Health and Disability Insurance asked the Belgian Health Care Knowledge Centre to explore how much health gain the entry of innovative oncology medicines has generated in real life. An analysis of Belgian observational data pertaining to 40 innovative oncology medicines for 12 indications over the 2004-2017 period found substantial increases in expenditure and treatment costs and limited gains in life expectancy for half of these indications, although such data do not allow to establish a causal relationship between these costs and health gains (Neyt et al., 2021).

### Sustaining innovation and access to oncology medicines

Multiple avenues can be pursued to balance innovation with affordable access to oncology medicines (Lopes et al., 2017), with a few ideas being discussed in this section. For instance, a series of focus group discussions with 13 Belgian participants (from the National Institute for Health and Disability Insurance, pharmaceutical industry, hospital pharmacy, medical profession and academia) emphasised the role of prevention and education; the use of precision medicines, biosimilars and generics; the application of managed entry agreements and the collection of real-world evidence; and a stricter assessment of oncology medicines within the context of marketing authorisation and reimbursement decisions (Van Dingenen and De Sadeleer, 2019). With respect to the latter, a policy paper called for a long-term dialogue between the National Institute for Health and Disability Insurance and the pharmaceutical company, with expedited marketing authorisation and conditional reimbursement based on the claimed therapeutic benefit of an oncology medicine and with re-assessment and potential revision of the reimbursement decision based on real-world evidence (Van Dyck et al., 2016).

To support the adoption of innovative oncology medicines in clinical practice, the European Organisation for Research and Treatment of Cancer has advocated the conduct of ‘treatment optimisation studies’ and has published an empirical framework for undertaking them (European Organisation for Research and Treatment of Cancer, 2020). Treatment optimisation studies are clinical trials that aim to optimise the way in which therapeutic interventions are applied in real-world settings (Saesen et al., 2020). However, more structural support is needed to explore how treatment optimisation studies can be implemented into the oncology medicine development paradigm (Saesen et al., 2020; Saesen et al., 2021). The European Medicines Agency has recently established the Cancer Medicines Forum in collaboration with the European Organisation for Research and Treatment of Cancer to investigate the feasibility of this implementation (Saesen et al., 2022).

## Market access of precision medicines

### Context and challenges

Precision medicine entails the use of a companion diagnostic test to detect the appropriate patient before administration of the medicine. Therefore, when considering the precision medicine for reimbursement, ideally the companion diagnostic should be considered simultaneously. This is because both entities contribute to the (cost-)effectiveness of the precision medicine treatment, this is also called ‘co-dependency’. Because of the way health care systems are structured throughout Europe, many countries are facing difficulties to translate this co-dependency into a joint assessment and reimbursement decision-making process (Wurcel et al., 2016). Also, criticism is expressed by diagnostic and pharmaceutical companies that novel *in vitro* diagnostic testing techniques are used in clinical practice that are out of the scope of current reimbursement codes for companion diagnostics in Belgium.

### Sustaining innovation and access to precision medicines

Belgium was one of the first countries in Europe to introduce a joint procedure for reimbursement of a companion diagnostic and its precision medicine at the level of the National Institute for Health and Disability Insurance (Govaerts et al., 2020), thus enabling joint access through the statutory health care insurance system. However, this joint procedure does not apply to immune-histochemistry tests, which are used to identify patients for immunotherapies (i.e. quantification of the PD-L1 expression levels).

Given that many precision medicines have obtained marketing authorisation in recent years, the number of patients who are eligible to be tested and companion diagnostic test expenditure have also increased. Therefore, the Belgian Minister of Health has stated the intention to develop an integrated model supporting the funding of the various components of precision medicine (Vandenbroucke, 2020).

In view of the use of reimbursement codes on companion diagnostics, Pharma.be (the Belgian umbrella organisation for the innovative pharmaceutical industry) and the Government are in discussions to address several issues with a view to support access to novel precision medicines in Belgium.

## Market access of orphan medicines

### Context and challenges

Orphan medicines have the connotation of being expensive. Given their rising share in national health care budgets and more orphan medicine candidates in the pipeline, this category of medicines raises concerns in terms of sustainability (Orofino et al., 2010; Schey et al., 2011). Although generic versions of orphan medicines have been developed that might decelerate orphan medicine expenditure, their adoption has not been evident (Di Paolo and Arrigoni, 2018). Today, 75.4% of orphan medicines are reimbursed in Belgium under managed entry agreements (National Institute for Health and Disability Insurance, 2020b). However, there is a lack of transparency on the appraisal process or decision criteria for reimbursement. Furthermore, delisting of orphan medicines that fail to meet pre-defined conditions at the end of the managed entry agreement is rarely implemented (Gerkens et al., 2017). In turn, highly mediatised cases such as those of baby Pia and Viktor may spark complex, ethical debates and increase pressure to provide reimbursement to an orphan medicine despite uncertainty regarding its effectiveness as a result of small and/or uncontrolled clinical trials (Picavet et al., 2013; Simoens et al., 2013). In turn, the lack of clinical evidence complicates the conduct of an economic evaluation (Simoens, 2011). As a result, the National Institute for Health and Disability Insurance may find it difficult to substantiate the allocation of limited resources to the reimbursement of orphan medicines.

### Sustaining innovation and access to orphan medicines

A number of avenues are discussed here for improving the Belgian reimbursement process for orphan medicines.

First, there is scope to further structure the reimbursement process, while still allowing flexibility. This can be done, for instance, by setting up a decision matrix which details all reimbursement criteria (including those which are currently considered in an implicit manner, such as ethical arguments) and against which an orphan medicine can be assessed for reimbursement purposes. In addition, by publishing this matrix, the National Institute for Health and Disability Insurance would increase transparency surrounding the reimbursement process, allowing understanding and acceptance of the final decision by the general public (Blonda et al., 2021).

Second, such a decision matrix also needs to consider the management of uncertainties in the evidence base. For this purpose, the so-called TRUST4RD instrument (Tool for Reducing Uncertainties in the evidence generation for Specialized Treatments for Rare Diseases) has been developed during a series of multi-stakeholder discussions set up by the National Institute for Health and Disability Insurance (Annemans and Makady, 2020). In particular, the authors suggest: 1) to identify and rank uncertainties during the development phase; 2) to match each evidence gap to an appropriate data source; 3) to document data issues; and 4) to find a solution that is reliable, reflective and respectful towards the multi-stakeholder team that is involved in this process. Simultaneously, an iterative dialogue should take place between the company and the National Institute for Health and Disability Insurance both pre- and post-launch.

Third, the Belgian authorities should support data collection and sharing by optimising rare disease registries set up by the European Reference Networks in collaboration with pharmaceutical industry (Heon-Klin, 2017; Tumiene et al., 2021). These rare disease registries can also be an instrumental source of data on cost and patient numbers in the context of budget impact analyses of orphan medicines. In Belgium (as in other countries), budget impact analyses of orphan medicines tend to be of low quality (Abdallah et al., 2021). More attention needs to be paid to conducting and reporting budget impact analyses for (orphan) medicines that adhere to good practice guidelines, such as those set up by the International Society for Pharmacoeconomics and Outcomes Research (Abdallah et al., 2021).

Fourth, the optimisation of uncertainty management and improved data collection would facilitate and improve the implementation of managed entry agreements (Annemans and Makady, 2020). Uncertainties need to be defined clearly and fully in order to enable efficient re-assessment of the orphan medicine after the expiry of the managed entry agreement. In addition, more resources need to be made available to support an ongoing dialogue with the company, to follow up on the evidence generated and its adherence to the conditions laid out in the managed entry agreement.

Fifth, there is a need to set up a decision-making and communication strategy that allows for the delisting of an orphan medicine if its managed entry agreement milestones have not been reached. Details of the assessment and the appraisal need to be logged and a summary provided to the public. Increased transparency and appropriate communication are crucial in enabling the National Institute for Health and Disability Insurance to delist an orphan medicine if the evidence does not support its further reimbursement and to re-invest the freed up budget into other cost-effective health technologies.

## Market access of advanced therapies

### Context and challenges

Advanced therapies cover a complex ensemble of gene-based, cell-based or tissue engineered products, with 14 advanced therapies having a valid marketing authorisation from the European Medicines Agency by April 2022, six of which are reimbursed in Belgium (pharma.be, 2022). Although the European Medicines Agency adopts several approaches to accelerate the approval of innovative advanced therapies such as conditional marketing authorisation, the Priority Scheme (PRIME), approvals under exceptional circumstances, accelerated assessments or compassionate use, market access remains a challenge (van Overbeeke et al., 2021). Whereas the European Medicines Agency focuses on the risk-benefit balance of medicines, health technology assessment agencies and payers consider cost-effectiveness and affordability in comparison with existing therapies to decide upon reimbursement. Here, European countries show heterogeneity in their choices, resulting in different levels of patient access between countries. On the one hand, long-term uncertainties related to safety and efficacy remain and are challenging to be tested in (relatively) short-term clinical trials (Tavridou et al., 2021). On the other hand, most indications for current advanced therapies are directed to rare diseases or a selected group of patients with high unmet needs, offering great promise for these patients. This factor, together with the existence of exclusivity rights on innovative advanced therapies, give companies power in price negotiations, with prices ranging from €300,000 to €2.3 million per treatment. Recent calls by patient families for crowdfunding even showed that (many) Belgian citizens seem to be willing to pay such high prices. Nevertheless, difficult choices are to be made in view of which advanced therapies (and other health technologies) can or need to be reimbursed.

### Sustaining innovation and access to advanced therapies

Several avenues to ensure a sustainable market access of advanced therapies are reported and need to be explored further (Kanavos et al., 2020; Picecchia et al., 2020; Simoens et al., 2022). First, early dialogues between developers, regulatory agencies and payers should become an obligatory step in the assessment procedure. Second, public-private partnerships in advanced therapy development should be stimulated or even mandated in certain disease domains, combining expertise and assets from both public and private sector entities such as industry, academia, patient organisations, regulatory and health technology assessment agencies, and payers. Upfront agreements on shared models for costs and risk sharing as well as future access to the advanced therapy product are needed. Third, increased partnerships between payers at the European level are to be organised, to align early dialogues and reimbursement strategies, and harmonise value assessment models for advanced therapies. Fourth, transparency on publication and communication of clinical trial results on advanced therapies is key to install trust at all levels of decision making. Fifth, innovative intellectual property based payment models should be considered, whereby intellectual property ownership may either be transferred, shared between the public-private partners or licensed out. Sixth, there is a need to further investigate how to design and implement outcome-based managed entry agreements with spread payments in the context of addressing the high budget impact and clinical uncertainties associated with advanced therapies. In Belgium, these and other avenues are currently being explored in a series of multi-stakeholder roundtable meetings on a market access pathway for advanced therapies (Inovigate, 2022).

## Market access of repurposed medicines

### Context and challenges

Repurposing existing medicines for new therapeutic indications (see also Box 1) has been put forward as an innovative treatment development strategy to address current medical needs. A well-known example is dimethyl fumarate: it was originally synthesised 50 years ago for the treatment of psoriasis and later developed by Biogen Idec for multiple sclerosis (Spencer, 2014). Repurposing is considered particularly useful to provide timely and affordable treatment options for rare and neglected diseases, for which commercial interests to develop new chemical entities are lacking (Hernandez et al., 2017; Scherman and Fetro, 2020). The key benefit of a repurposing strategy is the availability of an extensive body of knowledge and data for a candidate medicine (Bertolini et al., 2015). Conversely, it is also possible that there is insufficient data and this can pose a key challenge and increase the risk of failure in clinical trials and development costs.

BOX 1 **| Repurposing a medicine**

The term “medicine repurposing” covers several development scenarios. Medicine repurposing can refer to identifying new uses for experimental or investigational assets that went through several stages of clinical development (at least phase I clinical studies), but were “shelved” due to a lack of efficacy or commercial interest, or medicines that have been on the market but were withdrawn for commercial or other reasons. Pharmaceutical developers are increasingly interested in such a repurposing strategy, also called “medicine rescue,” as this involves fewer risks compared to developing new chemical entities and may create opportunities for new or additional intellectual property claims (i.e. patents for second and further medical uses) and regulatory exclusivities (Frail et al., 2015; Naylor et al., 2015). Furthermore, pharmaceutical developers often look for opportunities to identify new indications for medicines that are already authorised for one or more indication(s) and are still under basic patent or regulatory protection. Developing new uses for innovator products is often referred to as ‘life cycle management’ of the medicine, and may expand the patient population while delaying generic or biosimilar competition (Langedijk et al., 2016; Cha et al., 2018). Some repurposed medicines require product changes (e.g., change in dose, pharmaceutical form, route of administration) or are combined with other medicines or medical devices in the new indication. This can be a commercially interesting repurposing strategy as product changes may generate new intellectual property and enable a pharmaceutical developer to rebrand a product for its new use (Novac, 2013; Dilly and Morris, 2017). A final scenario covers the repurposing of approved medicines that are out of basic patent or regulatory protection and are used “as-is,” thus do not require any substantial product changes (Bloom, 2016).

Despite the potential benefits and the substantial increase in research and commercial activity in the field of medicine repurposing, the scientific and medical community is facing significant regulatory, legal and financial challenges (Verbaanderd et al., 2017; Breckenridge and Jacob, 2019). Challenges are especially apparent for authorised medicines that are out of basic patent and regulatory protection. Once the medicine loses its patent protection and/or regulatory exclusivity, pharmaceutical developers are no longer incentivised to invest in additional research and regulatory procedures for new indications because generic or biosimilar competitor products will enter and adapt their labels based on the reference product. Indeed, Langedijk et al. showed that 92.5% of extensions for new indications took place during the period of exclusivity granted to developers for new medicinal products authorised via the European Union centralised procedure (Langedijk et al., 2016). As a result, the development of off-patent products that do not require any product changes to differentiate them from competitors is often discontinued, even though promising evidence may exist to support a new use. This situation has led to off-patent repurposing candidates being called ‘financial orphans’ (Sukhatme et al., 2014).

Because return on investment is expected to be low, new therapeutic indications for approved, off-patent medicines are mainly studied in independent clinical studies initiated and led by researchers from academia, research institutes or collaborative groups (Pantziarka et al., 2018). However, these researchers typically do not have the knowledge, expertise, resources or intention to apply for and maintain a marketing authorisation, and to fulfil post-marketing responsibilities. Moreover, they often have little experience in designing registration trials, which have to meet strict regulatory requirements, and they may not have access to all relevant data concerning the medicine, in particular the non-clinical and clinical pharmacology data submitted as part of the original authorisation dossier. Engagement with the pharmaceutical industry could facilitate collection of the necessary data and the registration of the new indication. However, due to a lack of incentives and a lack of control over the quality of the data that is generated by third parties, marketing authorisation holders may be reluctant to get involved in medicine repurposing research.

### Sustaining innovation and access to repurposed medicines

In recent years, medicine repurposing has caught the attention from policy makers and regulators. In Europe, medicine repurposing became part of the agenda of the European Commission Expert Group for Safe and Timely Access of Medicines for Patients. This resulted in the establishment of the “Proposal for a framework to support not-for-profit organisations and academia (institutions and individuals) in drug repurposing” (European Commission Expert Group for Safe and Timely Access of Medicines for Patients, 2019). A pilot to test this framework was launched in October 2021 by the European Medicines Agency and the Heads of Medicines Agencies (European Medicines Agency, 2021c). Also, in February 2020, European experts in oncology published an awareness call for bringing new indications on-label for ‘old’ medicines (Rauh et al., 2020).

At the Belgian level, a number of initiatives have been taken to support medicine repurposing as well. For instance, the Belgian Health Care Knowledge Center has included medicine repurposing as a focus area in their calls for funding of independent clinical research (Belgian Health Care Knowledge Centre, 2021). The Anticancer Fund, a Belgian-based not-for-profit organisation, scientifically and financially supports independent clinical trials with off-patent or generic repurposed medicines in cancer patients.

Finally, several companies have specifically built their business model around finding and commercialising new uses for existing medicines. In Europe, the Value Added Medicines Group, a sector group of Medicines for Europe, focuses on adding value to known molecules by “finding a new indication (medicine repositioning), finding a better formulation or dosage (medicine reformulation), or developing a combined medicine regimen, adding a new device or providing a new service (medicine combination)” (Toumi and Remuzat, 2017). Additionally, several consulting firms are offering specific guidance to develop commercial medicine repurposing strategies.

## Market access of generic and biosimilar medicines

### Context and challenges

One of the ways to increase access to innovative medicines and keep our expanding health care budget under control is to stimulate a competitive off-patent medicines market (Dylst et al., 2013; IQVIA, 2018, 2020). In the small molecules market, generic medicines (European Commission, 2004) can introduce competition after the exclusivities have expired of originator therapeutics. For biological medicines, biosimilars (European Medicines Agency, 2014b) enter the market after loss of exclusivities as equivalent therapeutic options for originator biologicals. However, the current Belgian landscape for off-patent medicines is challenged by several factors. In the context of developing and sustaining the Belgian generics market, some efforts have been made already, including the introduction of a reference pricing system in 2011 and the implementation of policies targeting health care professionals (i.e. quotas of low-cost prescriptions, prescribing by international non-proprietary name) (Simoens et al., 2005; Dylst et al., 2013). Also for the off-patent biologicals market, a series of policy measures were put in place during past years in order to increase the competitiveness of the market (Dylst et al., 2014; Medaxes, 2020; Moorkens et al., 2020; Vandenplas et al., 2021). However, despite these efforts, generics and biosimilars still face delayed market access and have low market shares compared with other European countries, both in the retail and the hospital setting (Simoens et al., 2005; Medaxes, 2020, 2021; Vandenplas et al., 2021). Nonetheless, merely achieving high market shares of generics or biosimilars should not be the sole focus. One should look at the wider picture to guarantee a competitive and sustainable market for a whole therapeutic class of products.

In Belgium, generics are generally 54%–65% lower priced than the initial price of originators (Medaxes, 2020). Therefore, generic medicines constitute a considerable opportunity for health care authorities to obtain savings, while maintaining the standards of quality of care and expanding access to treatments (European Medicines Agency, 2007). In this context, and mainly due to mandatory price cuts introduced following the market entry of generics, savings of €1.9 billion have been achieved in 2020 (Medaxes, 2020). In addition to the off-patent small molecules market, the Belgian off-patent biologicals market also contributes to important savings for the National Institute for Health and Disability Insurance (IQVIA, 2020; Goeman et al., 2021; Vandenplas et al., 2021). As a result of biosimilar competition, list price decreases have reduced overall pharmaceutical spending with 5% across Europe, depending on the product class (IQVIA, 2020). In addition, confidential discounts through tender procedures have led to further savings for national health care systems (Barbier et al., 2021). In Belgium, a recent analysis with data from the National Institute for Health and Disability Insurance revealed that daily costs of biologicals decreased with over 32% after patent expiry (Goeman et al., 2021; Vandenplas et al., 2021).

These savings in the Belgian off-patent biologicals market have been realised mainly due to short-term cost containment policies. In particular, biosimilars enter the Belgian market at a lower price than their reference biological. In addition, reference products lower their prices as well due to mandatory price decreases when they have been reimbursed for over 12 years (National Institute for Health and Disability Insurance, 2019b). These measures lead to substantial savings, mostly regardless of biosimilar usage. Yet, one should not ignore the importance of biosimilars for a more sustainable health care system. If no biosimilar medicines would enter the Belgian market in the future, the Belgian health care system would miss out on several benefits. Competition in tenders would be lost, leading to higher net prices for hospitals and decreased savings for the health care system. We also know from the experience of various European countries that price competition can lead to additional savings in the ambulatory market (Autoriteit Consument & Markt, 2019; Moorkens et al., 2021). However, price competition requires a certain market volume of biosimilars. Also, pharmaceutical companies would no longer be challenged to engage in product innovation (i.e. new administration routes or more convenient package sizes) (Dutta et al., 2020). Moreover, in addition to their reference products, pharmaceutical companies also market new competing products for similar indications (i.e. JAK inhibitors as an alternative to off-patent tumor necrosis factor-alpha inhibitors). There is a risk that if biosimilars would not enter the Belgian market, the market will shift even further towards more expensive alternatives with limited added value in the future (Smolen et al., 2014; Ferrante and Sabino, 2019; Vandenplas et al., 2021).

In coming years, several new biosimilars are expected on the European markets due to exclusivity loss of originator biologicals. In Belgium, this group of biologicals had an aggregate cost of €831 million in 2019, which accounted for 15.9% of total pharmaceutical expenditure (National Institute for Health and Disability Insurance, 2020b; Goeman et al., 2021). Most new biosimilars are likely to emerge from the field of oncology (Barbier et al., 2020; Medaxes, 2021). If Belgium wants to exploit the potential of this new wave of biosimilars, it will have to develop and implement a sustainable policy framework as soon as possible.

### Sustaining innovation and access to generic and biosimilar medicines

In order to fully realise the benefits that generic medicines can bring to the Belgian health care system, a coherent and comprehensive policy that supports competitive off-patent markets with improved generic market shares is needed. According to Medaxes (the Belgian umbrella organisation for biosimilar and generic pharmaceutical industry), aspects such as the optimisation of the electronic prescribing system, together with measures to support the early market access of generics, can support competitive dynamics within the market (Medaxes, 2019).

Over the past years, Belgian policy makers launched various measures with the aim of increasing uptake of biosimilar medicines. In 2015, in the context of the Pharma Pact of the Future, a Convention was signed between the Ministry of Social Affairs and Health and the principal stakeholders, pledging to increase biosimilar use in Belgium. Agreements set out in the 2015 Convenant included the target of 20% biosimilar prescribing in bio-naïve patients, with a regular monitoring of hospitals to track usage (Moorkens et al., 2020). Following on from this, hospitals were urged to timely organise tenders and ensure a level-playing field for originator biologics and biosimilars. While an improvement in terms of uptake was noted for a selection of biosimilar products, overall biosimilar market shares remained low (Moorkens et al., 2020). At present, a more holistic policy approach is being strived for, with the launch of a “best-value biological’” programme in 2019. Rather than concentrating on initiatives that aim to maximise short-term savings or on one-off measures, the focus of the proposed policy framework is on reaching healthy competition between originator biologicals and biosimilars *via* an integrated set of policy measures (Moorkens et al., 2020; Vandenplas et al., 2020; Van Wilder, 2021). In preparation of the policy framework, the National Institute for Health and Disability Insurance assigned a best-value biologics programme manager to help navigate initiatives, launched a Biosimilar Task Force, and sponsored academic research to gain in-depth understanding of the Belgian off-patent and biosimilar market dynamics (National Institute for Health and Disability Insurance, 2018). The latter resulted in a report with detailed recommendations on how to move towards a level-playing field with increased competition from biosimilars. Recommendations of the report include optimising tender practices, installing risk-sharing mechanisms, continuing educational efforts towards health care professionals and patients, and pursuing temporary market share quota for biosimilars in the ambulatory care context (Vandenplas et al., 2020). Although biosimilar quota have been implemented with success in neighbouring countries, such a measure may be met with resistance from manufacturers of originator biologicals, but is likely to be required to stimulate the competitiveness of the market. While awaiting concrete governmental and policy actions, the General Council of the National Institute for Health and Disability Insurance has endorsed the report’s recommendations while inserting a “non-discrimination” condition (National Institute for Health and Disability Insurance, 2020a; Van Wilder, 2021).

Another avenue within a more holistic best-value policy framework is the re-assessment of cost-effectiveness of all products within the same therapeutic class at the time of biosimilar market entry. As biosimilar market entry may trigger price reductions, the cost-effectiveness of biologicals (and other innovative products) alters, warranting the need to evaluate reimbursement conditions beyond individual product level and to reconsider modalities within the whole therapeutic class (Simoens and Vulto, 2021; Vandenplas et al., 2021). In addition, shifts from off-patent biologicals to more expensive second-generation products or new therapeutic classes with limited added value contribute to sub-optimal spending of health care budgets and threaten the sustainability of the market. Besides existing instruments such as a ‘group revision’, new approaches are needed to address this (Vandenplas et al., 2021).

## International perspective

Balancing access to innovative therapies while ensuring financial sustainability of the health care system remains a challenge for many countries, as illustrated for Belgium. For example, the European Commission’s “Pharmaceutical Strategy for Europe” aims, amongst other things, to support investment in R&D of innovative medicines and to promote competition in the off-patent medicines market (European Commission, 2020). Emerging markets such as Brazil, China, India, Indonesia, Mexico, Russia, South Africa and Turkey will face the same challenge as pharmaceutical (and medical device) R&D and innovation is increasingly taking place in these countries, while the demand of their populations for health care is growing (Jakovljevic et al., 2017; Jakovljevic et al., 2021; Jakovljevic et al., 2022a; Jakovljevic et al., 2022b; Sapkota et al., 2022). At global level, an analysis of medicine expenditure in a representative sample of 11 countries showed that spending growth as a result of innovation in oncology and immunology has been offset by savings and increased patient access due to the market entry of generic and biosimilar medicines (Aitken et al., 2021).

The diverse initiatives taken, ongoing, planned and yet to be foreseen to improve access to medicines in Belgium as discussed above are also being considered or implemented in other countries (Vogler et al., 2018). Although a comprehensive discussion of international trends in sustainable market access to medicines falls outside the scope of this *Perspective* and a one-size-fits-all approach may not exist for all countries, some concepts seem to be key. These include: horizon scanning, price transparency, regulatory collaboration, managed entry agreements, real-world data collection and use.

First, horizon scanning is essential in preparing health care systems for the sustainable market access of new medicines (World Health Organization, 2015; Godman et al., 2018). A number of countries have set up national or more recently international horizon scanning activities and their experience highlights the importance of not setting up horizon scanning as a stand-alone activity, but as part of an integrated system to manage access, pricing and reimbursement of new medicines (Vogler, 2022).

Second, the Organisation for Economic Co-operation and Development and others have advocated price transparency in medicines markets (OECD, 2018; Hagenbeek et al., 2020). This pertains to how medicine prices are set by pharmaceutical industry, which net prices are negotiated between a health care payer and a company in the context of a managed entry agreement, which and how medicine pricing (and reimbursement) criteria are applied by a country. Although there are sound theoretical arguments in favour of transparency of medicine prices, a recent report of the European Observatory on Health Systems and Policies concluded that the impact of price transparency on innovation and sustainable access to medicines in practice remains unclear (Webb et al., 2022).

Third, there is a need to strengthen regulatory collaboration between countries. To this effect, evidence requirements need to be further streamlined between regulatory authorities of different countries and between regulatory authorities involved in different stages of the drug life cycle. Initiatives such as the proposed African Medicines Agency with respect to the former and the collaboration between the European Medicines Agency and EUnetHTA with respect to the latter are to be welcomed. Furthermore, countries can augment their buying power by collaborating through regional medicine procurement schemes (Vogler et al., 2021) such as the Strategic Fund of the Pan American Health Organization (Pan American Health Organization, 2022).

Fourth, managed entry agreements are increasingly used across the world to manage clinical and/or budgetary uncertainties associated with innovative medicines (Annemans et al., 2011; Zampirolli Dias et al., 2020). Arguably, countries apply such agreements in an *ad hoc* manner, and the type of agreement for a specific medicine can differ between countries (Pauwels et al., 2017). Also, there may be changes in the managed entry approach over time, with Italy, for example, moving away from outcome-based agreements to financial-based agreements. Hence, decision makers would benefit from research that indicates which type of managed entry agreement needs to be used under which circumstances (e.g. dependent on type of health care system, medicine, risks) (Goodman et al., 2022; Whittal et al., 2022).

Fifth, real-world data collection and use throughout the drug life cycle has gained traction in recent years. Today, many health care systems are exploring the opportunities and addressing the challenges of integrating such data in market access decision making for medicines in general and their role in comparative effectiveness studies, health technology assessment and managed entry agreements in particular (Makady, 2018). There is room for further methodological guidance and identification of best practices to optimise real-world data collection and use.

## Data Availability

The original contributions presented in the study are included in the article/Supplementary Material, further inquiries can be directed to the corresponding author.
